# Deletion of Dystrophin In-Frame Exon 5 Leads to a Severe Phenotype: Guidance for Exon Skipping Strategies

**DOI:** 10.1371/journal.pone.0145620

**Published:** 2016-01-08

**Authors:** Zhi Yon Charles Toh, May Thandar Aung-Htut, Gavin Pinniger, Abbie M. Adams, Sudarsan Krishnaswarmy, Brenda L. Wong, Sue Fletcher, Steve D. Wilton

**Affiliations:** 1 Western Australian Neuroscience Research Institute, Perth, Australia; 2 University of Western Australia, Perth, Australia; 3 Centre for Comparative Genomics, Murdoch University, Perth, Australia; 4 School of Anatomy, Physiology and Human Biology, University of Western Australia, Perth, Australia; 5 Department of Paediatrics, Department of Neurology, Cincinnati Children’s Hospital Medical Center, Cincinnati, Ohio, United States of America; Rutgers University -New Jersey Medical School, UNITED STATES

## Abstract

Duchenne and Becker muscular dystrophy severity depends upon the nature and location of the *DMD* gene lesion and generally correlates with the dystrophin open reading frame. However, there are striking exceptions where an in-frame genomic deletion leads to severe pathology or protein-truncating mutations (nonsense or frame-shifting indels) manifest as mild disease. Exceptions to the dystrophin reading frame rule are usually resolved after molecular diagnosis on muscle RNA. We report a moderate/severe Becker muscular dystrophy patient with an in-frame genomic deletion of *DMD* exon 5. This mutation has been reported by others as resulting in Duchenne or Intermediate muscular dystrophy, and the loss of this in-frame exon in one patient led to multiple splicing events, including omission of exon 6, that disrupts the open reading frame and is consistent with a severe phenotype. The patient described has a deletion of dystrophin exon 5 that does not compromise recognition of exon 6, and although the deletion does not disrupt the reading frame, his clinical presentation is more severe than would be expected for classical Becker muscular dystrophy. We suggest that the dystrophin isoform lacking the actin-binding sequence encoded by exon 5 is compromised, reflected by the phenotype resulting from induction of this dystrophin isoform in mouse muscle *in vivo*. Hence, exon skipping to address DMD-causing mutations within *DMD* exon 5 may not yield an isoform that confers marked clinical benefit. Additional studies will be required to determine whether multi-exon skipping strategies could yield more functional dystrophin isoforms, since some BMD patients with larger in-frame deletions in this region have been reported with mild phenotypes.

## Introduction

Duchenne and Becker muscular dystrophy (DMD and BMD) are X-linked recessive muscle-wasting diseases arising from mutations in the massive dystrophin gene (*DMD*) that leads to degeneration of muscle. DMD is the most prevalent and severe form of childhood muscle wasting with a cited incidence of 1 in 3,500 live-male births, whereas BMD is a clinically milder form of the disease with slower disease progression [1, 2]. BMD mutations may present with a spectrum of phenotypes, depending upon the nature and location of the *DMD* lesion, as BMD mutations do not usually disrupt the reading frame, thereby allowing production of an internally shortened but functional dystrophin. BMD patients remain ambulant until at least 16 years of age but, in some mild or asymptomatic cases, may only be diagnosed by accident or late in life [3, 4]. Conversely, intermediate muscular dystrophy has been used to describe “mildly affected” DMD patients (those whose genetic structure would predict prematurely truncated dystrophin and loss of ambulation by 12 years of age) or “severely affected” BMD cases (those who would be expected to produce some functional dystrophin and therefore remain ambulant after 16 years of age). In 1989, Dubowitz stated that ‘Intermediate DMD patients are defined as patients with a dystrophinopathy with onset of symptoms (of motor difficulties) by about 5 years of age, similar to the classical DMD patients but with a slower rate of disease progression, with loss of ambulation between 13 and 16 years of age.” [5]. Children of the same age vary widely in clinical presentation, and patients that appear to have a milder dystrophinopathy have also been termed *"outliers*.*"* [6]

In over 90% of DMD cases, correlation between the disease phenotype and the genotype is obvious. However, there are exceptions to the reading frame rule, where an in-frame deletion may result in a severe phenotype, or conversely, some out-of-frame gene rearrangements or nonsense mutations present with relatively mild symptoms, consistent with a diagnosis of BMD [7, 8].

The pathogenic basis of particular in-frame dystrophin deletions reflects the number of exons lost, where deletions of 34 or more exons are usually associated with severe pathology [9], or secondary effects on pre-mRNA processing. Other in-frame deletions may have severe consequences, due to the loss of a crucial functional domain within dystrophin, eg the actin or beta-dystroglycan binding regions. Mutations in the 5’ region of the *DMD* gene frequently manifest as exceptions to the reading frame rule [10], and various mechanisms have been proposed to impact on the consequences of these mutations, including re-initiation of translation [11] and splicing perturbations [12].

Gualandi and colleagues reported that the loss of *DMD* exon 5 compromised pre-mRNA processing and selection of exon 6, consistent with a severe dystrophic phenotype [13]. Here, we describe another patient carrying a genomic deletion of *DMD* exon 5 who manifests with moderate/severe a phenotype, despite detectable dystrophin as demonstrated by immunofluorescence. We show that the *DMD* transcript from this patient is missing only exon 5, and the genomic loss of this exon does not obviously alter the recognition and splicing of exon 6.

Only a small number of patients missing *DMD* exon 5 have been described, therefore opportunities to explore phenotypic variation and perhaps understand the basis for more severe than expected disease are limited. In order to further evaluate a dystrophin isoform lacking the actin binding domain encoded by exon 5, we induced a transient dystrophinopathy model by skipping *Dmd* exon 5 in wild-type mice. Analysis of dystrophin, components of the dystrophin-associated glycoprotein complex, muscle architecture and isolated muscle function, reveals moderate dystrophic changes in diaphragm, intermediate between *mdx* and wild-type phenotypes. Excision of *Dmd* exon 5 greatly reduced dystrophin expression in mouse diaphragm and compromised muscle function.

Targeted exon skipping is emerging as a promising therapy to treat DMD, and at this time, the focus has been on exclusion of exons that flank frame shifting-deletions in order to re-frame the transcript. Similarly, removing an in-frame exon carrying an intra-exonic protein truncating mutation [14, 15], or a duplicated in-frame exon [16], would allow synthesis of functional dystrophin isoforms. The rationale is that re-framing the *DMD* transcript will produce a BMD-like isoform and reduce disease severity. Examination of BMD phenotype:genotype correlations can indicate *DMD* transcript structures most likely to generate functional dystrophin isoforms and therefore inform exon skipping strategies [17, 18]. We propose that removal of *DMD* exon 5 alone is unlikely to provide significant clinical benefit, and alternative exon skipping strategies or treatments should be considered for dystrophinopathy patients with mutations within exon 5, or those with duplications of exon 5.

## Materials and Methods

### Patient clinical summary

The patient showed normal development until 2^nd^ grade. He had slightly enlarged calves and also presented with an unusual muscle atrophy involving the paraspinal muscles and quadriceps. DNA testing revealed a *de novo* in-frame deletion of dystrophin exon 5. A muscle biopsy was performed and the tissue analysed by a diagnostic laboratory for the expression of dystrophin and associated proteins. DNA testing revealed a *de novo* in-frame deletion of dystrophin exon 5.

### Cell culture and MyoD mediated myogenic conversion of fibroblasts

De-identified patient and normal human skin fibroblasts were obtained after informed consent (University of Western Australia Human Research Ethics Committee approval RA/4/1/2295). The fibroblasts were cultured in DMEM (Life Technologies, Melbourne, Australia) supplemented with 20% foetal calf serum (FCS) (Serana, Bunbury Australia), 1% GlutaMax^™^-I (Gibco Life Technologies, Melbourne, Australia), 10 U/mL penicillin (Invitrogen), 10 mg/ML streptomycin (Life Technologies) and 250 ng/mL amphotericin B (Sigma-Aldrich, Sydney, Australia). Fibroblasts were transformed into the myogenic lineage by transfection with an adenoviral construct expressing MyoD (*Ad5*.*f5*.*0*.*AdApt*.*MyoD*) at a multiplicity of infection of 200 (The Native Antigen Company, Oxford, UK) [19]. These cells were then differentiated in low serum media (5% HS-DMEM) for 3–4 days as described by Harding *et al* [20]. The 24 well plates were pre-coated with 50 μg/mL poly D-lysine (Sigma) and 100 μg/mL Matrigel (VWR, Brisbane, Australia) for 1 hour each, respectively. The patient and normal fibroblasts were seeded at 3 x 10^4^ cells/well in the pre-prepared 24 well plates.

### Splice switching antisense oligomers

An antisense oligomer, composed of 2’-O-methyl modified bases on a phosphorothioate backbone, was synthesized on an Expedite 8909 Nucleic Acid synthesizer using reagents from Azco Biotech (Ca, USA). Designed to induce *DMD* exon 6 skipping, the AO (5’-UAC GAG UUG AUU GUC GGA CCC AG-3’) annealed to bases 69–91 in exon 6, and had been shown to induce specific exon 6 skipping [21].

A panel of splice switching AOs was developed to excise mouse dystrophin exon 5, targeting similar coordinates used to excise human dystrophin exons 5 (Table 1). The 2’-O-methyl modified oligomer 5’ UAU GAU UUC CAU CCA CUA UGU CAG UGC UUC 3’ (Table 1, underlined) annealing to bases 20–49 in mouse dystrophin exon 5, was identified to efficiently remove the target exon 5 *in vitro*. This sequence was synthesized as a phosphorodiamidate morpholino oligomer coupled to the cell penetrating peptide *k* [22] (PPMO) for *in vivo* experiments.

**Table 1:** Sequence of splice switching 2’-O-methyl modified AOs to induce skipping of mouse *Dmd* exon 5. The underlined sequence (M5A(+20+49) was synthesised as a phosphorodiamidate morpholino oligomer coupled to the cell penetrating peptide *k* [22].

AO	Sequence (5’-3’)
**M5A(-03+26)**	GUG CUU CCU AUA UUC ACU AAA UCA ACC UG
**M5A(+20+49)**	UAU GAU UUC CAU CCA CUA UGU CAG UGC UUC
**M5A(+30+59)**	AGA GUG AGU UUA UGA UUU CCA UCC ACU AUG
**M5A(+35+65)**	AAA CCA AGA GUG AGU UUA UGA UUU CCA UCC A
**M5A(+40+69)**	AAU CAA ACC AAG AGU GAG UUU AUG AUU UCC

### Transfection of myogenic cells, RNA extraction and RT-PCR

The myogenic cells were transfected, 3 days after the initiation of myogenic differentiation, with the 23mer AO complexed with Lipofectamine 2000^®^ (1:1 w/w) in Opti-MEM media (Gibco) as per the manufacturer’s instructions. AO concentrations of 2.5, 5, 10, 25, 50 and 100 nM were used and the cells were then incubated at 37°C for 3 days before RNA extraction.

The total RNA was extracted using Trizol^®^ (Life Technologies) according to the manufacturer’s instructions and the RNA pellet resuspended in 30 μL of sterile water (Baxter Healthcare, Australia). Approximately 100 ng of total RNA was used as template for the initial reverse transcriptase PCR reaction (55°C for 30 minutes), using Superscript^®^ III One-step RT-PCR (35 cycles) of 94°C, 55°C and 72°C for 0.5, 1 and 2 minutes respectively to amplify *DMD* exons 1 to 10. A 1 μL aliquot from the primary reaction was then used as template for nested PCR using AmpliTaq Gold (Applied Biosystems, Melbourne, Australia) amplifying across *DMD* exons 1 to 10 with the inner primer set for 30 cycles. Obtained from (Geneworks, Thebarton, Australia), the outer and inner primer sets spanning *DMD* exons 1–10 described by Roberts *et al*. [23] were used for the amplifications. Forced myogenic conversion of the fibroblasts is not an efficient process and whilst adequate for RNA studies, the degree of myogenic differentiation of the cultures makes detailed protein studies challenging.

### Deletion breakpoint mapping in DMD introns 4 and 5

Normal and patient fibroblasts were propagated, trypsinised and genomic DNA harvested using the Pure Link^™^ Genomic DNA kits (Life Technologies). PCR primers were designed to generate amplicons of approximately 3kb across introns 4 (~21.4 kb) and 5 (~6.6kb) to localize the breakpoints in the flanking introns. Long range PCR was performed on ~60 ng of genomic DNA using TaKaRa LA Taq polymerase (TAKARA Biotechnology (Dalian) Co., LTD, Japan) with LA and GC rich amplification buffer according to the manufacturer’s instructions. Primer sequences are shown in Table 2. The PCR amplicons from the normal and exon 5-deleted dystrophin genes were fractionated on 2% agarose gels and the images captured on a Fusion FX gel documentation system (Vilber Lourmat, Marne-la-Vallée, France). The approximate locations of breakpoints in introns 4 and 5 were identified by comparison of amplification product sizes, generated from normal and patient DNA. Additional primers (Table 3) were designed to sequence the intron 4:5 junction using BigDye sequencing chemistry, with analysis by the Australian Genome Research Facility, Perth, Australia.

**Table 2:** Primers used for long range PCR to amplify *DMD* exon 4 to exon 6 from genomic DNA to identify the patient’s deletion breakpoint. The predicted amplicon size is estimated according to the reference sequence NG_012232.1.

Primer	Sequence (5’-3’)	Detected in Patient	Amplicon size* (bp)
HDEx4F	AAT GTC AAC AAG GCA CTG CGG	Yes	3039
HDIn4 R1	TAG GCC AGT ATT TCT TCA ACA GGT		+c223—c264+3000
HDIn4 F1	ACC TGT TGA AGA AAT ACT GGC CTA	Yes	3000
HDIn4 R2	TGG AGA GAA TAT GGA GAA ATA		c264+2977 to c264+6000
HDIn4 F2	TAT TTC TCC ATA TTC TCT CCA	Yes	3000
HDIn4 R3	GAG GAG ACC CTA AGG AAA AGA G		c264+5980 to c264+9000
HDIn4 F3	CTC TTT TCC TTA GGG TCT CCT C	Yes	3060
HDIn4 R4	CCA TTG AGA GAT GCA TGT ATT GAA TC		c264+8979 to c265-9378
HDIn4 F4	GAT TCA ATA CAT GCA TCT CTC AAT GG	No	3060
HDIn4 R5	GTT CTC TCT GAA TAC AGA TTT		c265-9402 to c265-6301
HDIn4 F5	AAA TCT GTA TTC AGA GAG AAC	No	2986
HDIn4 R6	GAG GAG GGT TAG GCA GAT GTT		c265-6320 to c265-3314
HDIn4 F6	AAC ATC TGC CTA ACC CTC CTC	No	3060
HDIn4 R7	GGT CAA CTG GAG TAT CTC C		c265-3335 to c265-241
HDIn4 F7	GGA GAT ACT CCA GTT GAC C	No	2933
HDIn5 R1	AGA TGG AGT TGG AAA GGG CTT		c265-259 to c357+2751
HDIn5 F1	AAG CCC TTT CCA ACT CCA TCT	No	3060
HDIn5 R2	TGA ATG CAG TGA CGC TTC AT		c357+2733 to c358-841
HDIn5 F2	ATG AAG CGT CAC TGC ATT CA	Yes	1013
HDEx6R	GTT GAT TAC ATT AAC CTG TGG		c358-860 to c472

**Table 3:** Sequencing primers used in breakpoint mapping.

Primer	Sequence (5’-3’)
HDIn4F4a	TCC AAG TTA AAA CCG CAA ATG C
HDIn4F4b	AGA GTA CCT GGA AGT AAA TGC
HDIn4F4c	GTA ATC ATT CAC TTC ACA CAG
HDIn5R2a	GCT TGG CCA GTC TGC CCT CTA T
HDIn5R2b	GCT GCA AGT TGT CTA TGG GGT T
HDIn5R2c	GTA CAT CCC TTG AAT TGT CCA G

### Agarose gel electrophoresis

PCR products were resolved on 2% agarose gels in 1xTAE buffer as indicated, and the images captured using the Vilber Lourmat Fusion FX gel documentation system.

### Transient induction of a ‘*Dmd* exon 5 deletion’ mouse model

All experiments performed on animals were approved by the University of Western Australia Animal Experimentation Committee (approval number RA4/100/702) and carried out in strict accordance with the recommendations in the National Health and Medical Research Council *Australian code for the care and use of animals for scientific purposes 8th edition (2013)*.

A phosphorodiamidate morpholino oligomer conjugated to a cell penetrating peptide (5-PPMOk) [22] targeting dystrophin exon 5; M5A(+20+49), (5’ UAU GAU UUC CAU CCA CUA UGU CAG UGC UUC 3’) was injected via the intraperitoneal route into C57BL/10ScSn (C57BL/10) mice (n = 5) to induce a dystrophin isoform missing the amino acids encoded by exon 5. The *5-PPMOk* peptide conjugate, supplied by AVI Biopharma Inc. Bothell, Oregon, (now Sarepta Therapeutics) was re-suspended in sterile normal saline and pre-warmed to 37°C before injection [24, 25]. Over an 8-week period, beginning at 3–4 days of age, the C57BL/10 mice were given twice weekly intraperitoneal (IP) injections of *5-PPMOk* at 20 mg/kg. Mice assigned to the control groups, C57BL/10 (n = 5) and C57BL/10ScSn*Dmd* ^mdx^ (*mdx)* mice (n = 4), received sham treatment containing an equal volume of normal saline. The mice were anaesthetised (IP, sodium pentobarbitone 40mg/kg) a week after completing the treatment regimen, sampled for physiological testing and then killed via cervical dislocation before further analysis. Diaphragm muscles were snap-frozen in liquid nitrogen-cooled isopentane and stored at -80°C for molecular, histological and immunofluorescence studies.

End point analysis and tissue sampling was performed under sodium pentobarbital anesthesia, and all efforts were made to minimize suffering.

### Western blot analysis

Approximately 30 mg of tissue was homogenized in 600 μl of 125 mM Tris-HCl (pH 6.8), 15% SDS (w:v), 10% glycerol (v:v), 0.5 mM phenylmethylsulfonyl fluoride and 1 μl protease inhibitors cocktail (Sigma Aldrich, Sydney, Australia) per 9μg of tissue. Western blots were carried out essentially as described by Cooper [26]. Briefly, 5 μl of diluted aliquots of protein extract (7.5μg total protein) were separated on NuPAGE 4–12% Bis-Tris gels (Life Technologies, Mulgrave, Australia) and stained with 0.2% Coomassie blue and destained with 0.7% acetic acid. Gel densitometry was used to estimate relative myosin expression to ensure equal protein loading on subsequent gels for western blotting.

Samples (5 μl containing 7.5μg total protein per lane) were loaded onto a 3% - 10% gradient SDS–polyacrylamide electrophoresis (PAGE) gel with a 3% stacking gel and run at 30 mA for 2h, with C57BL/10ScSn and *mdx* muscle samples used as positive and negative controls respectively. Fractionated proteins were then electro-blotted onto a nitrocellulose membrane (Amersham Pharmacia Biotech, Sydney, Australia) overnight at 290 mA in transfer buffer (25 mM Tris-HCl, pH 8.3, 192mM glycine, 20% methanol, 0.075% SDS). The transfer was performed with cooling water circulating at 18°C. Dystrophin and beta-dystroglycan were detected by probing the membrane with NCL-DYS2 monoclonal anti-dystrophin (1:100 Novocastra Laboratories, Newcastle-Upon-Tyne, UK) and beta-dystroglycan primary monoclonal antibody (1:4000 Santa Cruz Biotechnology), respectively. The signals were developed using WesternBreeze^®^ Chemiluminescent protein detection Kit (Life Technologies). Immunodetection was performed according to the manufacture’s protocol. Images were obtained using a Vilber Lourmat Fusion FX Gel document system and band intensities determined using the Bio-1D software (Vilber Lourmat). The band intensities obtained from the treatment groups were analysed and compared to the positive and negative controls.

### Histology and immunostaining on muscle

Diaphragm samples from sham treated *mdx* and C57BL/10 control mice, and the *5-PPMOk* treated C57BL/10ScSn were snap frozen in liquid nitrogen-cooled isopentane. Immunochemistry was performed on unfixed serial cryosections (7μm thick) from all the mice, stained with either monoclonal or polyclonal antibodies diluted in PBST containing 10% normal goat serum. Collagen staining with picro-sirius red is based on the method described by Puchtler, 1973 [27].

The following antibodies were used: monoclonal antibodies, NCL-DYS2 (Novocastra), against dystrophin carboxy terminal domain, (NCL-MHCd) (Novocastra) against developmental myosin heavy chain, and the polyclonal rabbit antibodies, AB 782259 (1:300, Santa Cruz) against anti-beta-dystroglycan and AB 310722, anti-neuronal nitric oxide synthase (nNOS) (1:1000, Millipore Upstate). Monoclonal antibody detection was performed using the Zenon Alexa fluor 488 labelling kit (Life Technologies) according to the manufacturer’s recommendation, without fixation, until after immunofluorescence staining was completed. Both NCL-DYS2 and NCL-MHCd primary antibodies were used at 1:10 dilution with a molar ratio of 4.5:1. Mouse IgG1 anti Aspergillus niger cytochrome oxidase (DakoCytomation code X093101) (AB 577451) was used as an isotype negative control antibody (data not shown).

Goat-anti-rabbit IgG Alexa Fluor 594 (1:400) (Molecular Probes) was used as secondary antibody to detect the rabbit polyclonal antibodies against beta-dystroglycan (1:300) and neuronal nitric oxide synthase (1:1000). Cryosections were incubated at room temperature for 1.5 hours before washing in PBST and incubation with the secondary antibody at room temperature for an hour, washed in PBST and mounted under a coverslip in anti-fade aqueous mountant after washing in PBS. As above, negative controls were prepared by substituting the primary antiserum with PBST containing rabbit immunoglobulin fraction (DakoCytomation X0936), diluted to match final immunoglobulin concentration on duplicate serial sections. An Olympus IX-70 inverted microscope was used to examine the sections. Images were captured on an Olympus DP70 digital camera (Olympus Australia Pty Ltd, Sydney, Australia) and photographed at 10x and 20x magnification. Each set of serial sections were photographed in batches and exposed under the same settings to allow valid comparison.

## Results

### Clinical details

#### Clinical summary at presentation, age 9 ½ years

The patient, aged 9-years, 6-months was noted at around 5 years of age to have enlarged calves, associated with a slow run, difficulty climbing stairs and using a Gower’s manoeuvre to rise from the floor. Around 6 years of age concerns regarding his inability to keep up with his peers were communicated to his parents and he was referred for further investigation. His CK was elevated (19,000 U/Dl), genetic testing showed a deletion of *DMD* exon 5 and he was diagnosed with Becker’s muscular dystrophy at the age of 8-years. He had a baseline cardiac evaluation with normal echocardiogram and pulmonary evaluation.

Although the patient has an in-frame deletion of *DMD* exon 5 that predicts a Becker’s muscular dystrophy diagnosis, his signs and symptoms of prominent pelvic girdle weakness (full timed Gower’s in 3.2 seconds); plantigrade stance; run with increased upper extremity swings and a waddle; unable to extend his hips fully against gravity for the table top manoeuvre; rising from a chair by turning his body, followed by hand push off the chair and waddling gait with lordosis with a heel-toe progression warranted further investigation. The diagnostic laboratory undertook analysis of limb girdle muscular dystrophy (LGMD) genes and found no sequence variants for *CAPN3*; the autosomal recessive LGMD genes, *FKRP*, *DYSF*, *TCAP*, *TRIM32*, *TTN*, *SGCA*, *SGCB*, *SGCE* and *SGCG*. A heterozygous change was found in *ANO5* exon 15 (undocumented variant of uncertain clinical significance).

The patient phenotype was considered to be more consistent with a severe Becker muscular dystrophy and the diagnosis was revised accordingly at age nine and a half years. In view of the findings of the more severe phenotype, steroid therapy was recommended.

The patient profile since diagnosis of his *DMD* exon 5 deletion is summarised in Table 4.

**Table 4:** *DMD* exon 5 deletion patient data obtained at diagnosis and follow up visits.

	Diagnosis	Follow up	Recent visit
Age	9.5 years	13 years	15.25 years
CK	19000		
FVC	118%		
Gower’s	3.2 s	5.9 s	11.7 s
Run 30ft	4.8 s	6.6 s	7.93 s
4 step climb	-	3.7 s	4.7 s

#### Histology and dystrophin analysis

**Fig 1:**
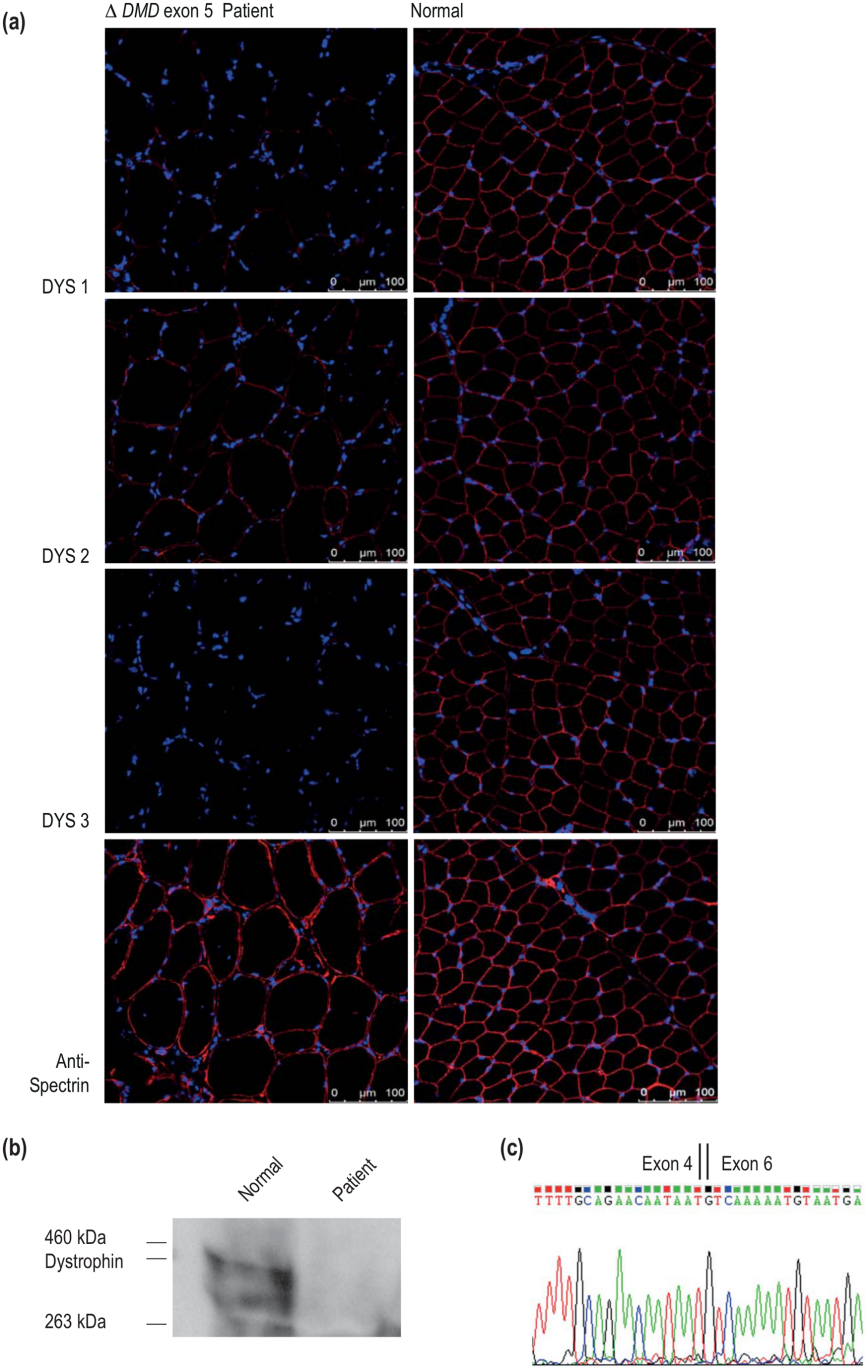
Dystrophin and spectrin expression in patient and normal muscle. **(a)** Dystrophin immunofluorescence using Novocastra Laboratories antibodies, NCL DYS-1 (upper row, specific for the rod domain) NCL DYS-2 (upper middle row, specific for the carboxy-terminus) and NCL-DYS 3 (lower middle row, specific for the amino-terminus) and spectrin (lower row) on patient (left panel) and normal human muscle (right panel) cryosections. Nuclei are stained with DAPI. (Bar = 100μm). (b) Western blot using NCL-DYS 1 to detect dystrophin in normal and patient muscle sample. (c) Sequencing of the *DMD* RT-PCR exon 1–10 amplicon from the exon 5-deletion patient showing the junction of *DMD* exons 4 and 6.

A muscle biopsy from the patient was analysed and indicated dystrophic changes, including fibre size variation. Immunofluorescence using NCL-DYS 1 (dystrophin rod domain) and DYS-2 (carboxy-terminus) showed reduced intensity, while that for NCL-DYS 3 (amino-terminus) was almost undetectable (Fig 1a). Very low levels of dystrophin were detected by western blot, less than 10% relative to the level in normal healthy muscle (Fig 1b). Spectrin immunostaining of the sarcolemma (Fig 1a) shows muscle fibres of irregular size, with some very large diameter fibres and many mononuclear cells in the patient section, while normal muscle section shows muscle fibres of very regular size and shape, typical of healthy muscle. The diagnostic laboratory reported that the staining intensity for alpha-sarcoglycan, merosin, alpha-dystroglycan, collagen VI, dysferlin, caveolin, lamin A/C and emerin appeared normal (data not shown).

#### Confirmation of *DMD* exon 5 deletion and breakpoint mapping in patient cells

RT-PCR across dystrophin exons 1–10 on total RNA extracted from patient myogenic cells indicated that the patient *DMD* transcript is missing only exon 5. Sequencing of the dystrophin RT-PCR product confirmed the loss of exon 5, with precise splicing of exon 4 to exon 6 (Fig 1c).

**Fig 2:**
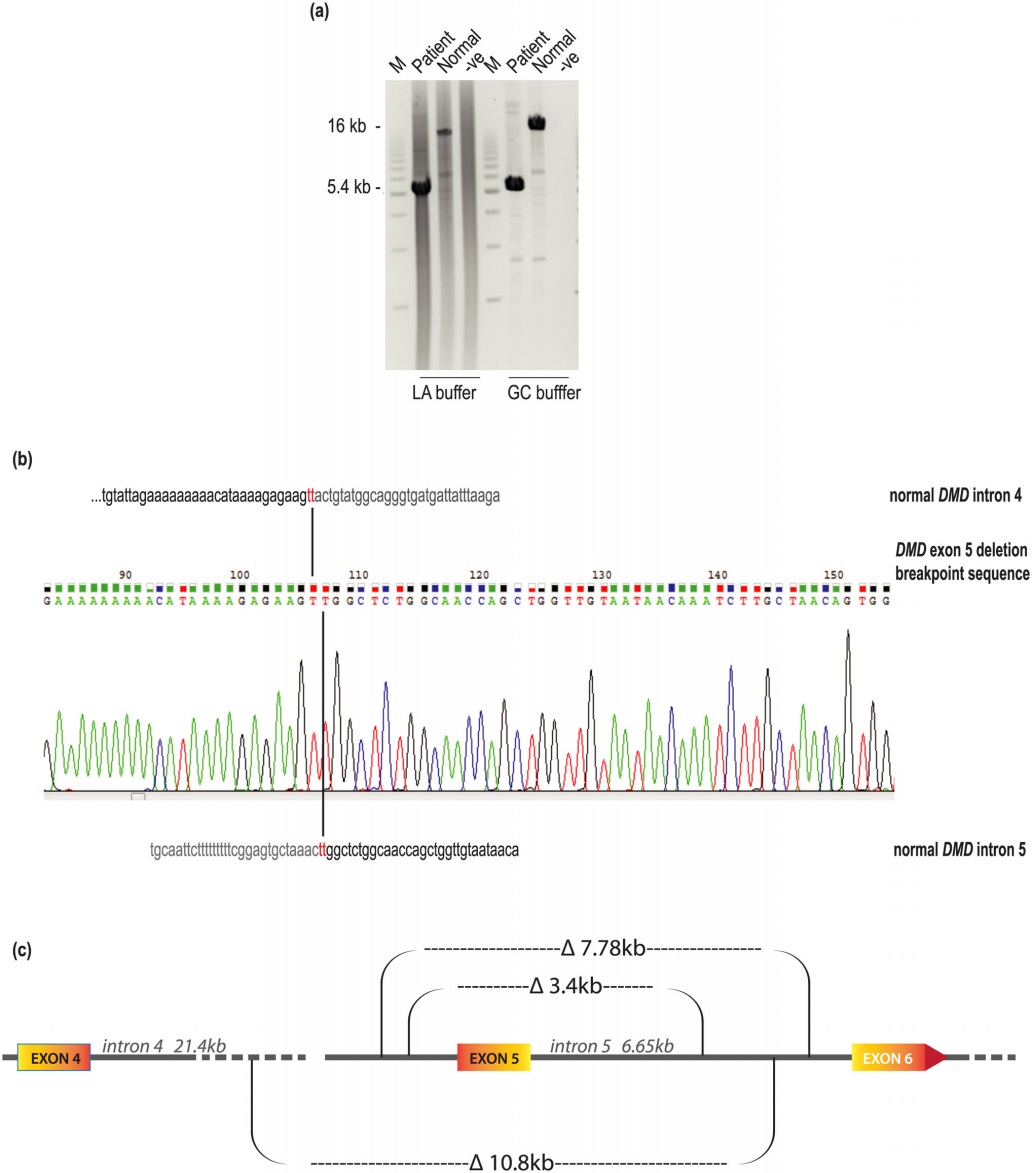
*DMD* deletion breakpoint characterization. (a) Amplification of patient and normal DNA with HIn4 F4 and the reverse primer targeted to exon 6 (HEx6ROuter), across the predicted *DMD* breakpoint location. A 1 kb DNA ladder was used as a marker to estimate amplicon sizes. (b) Alignment of nucleotide sequences from introns 4 and 5 showing the precise deletion breakpoint in the *DMD* exon 5 deletion patient. The size of the deletion is estimated, relative to the reference sequence NG_012232.1. (c) Representation of three *DMD* exon 5 deletions, showing deletion breakpoints. The patient described in the current study has the largest genomic deletion, 10.8 kb and the breakpoint downstream of exon 5 is located between the breakpoints in previously reported individuals, with 3.4 and 7.78 kb deletions [13].

The deletion breakpoints in introns 4 and 5 were identified by generating 3 kb amplicons across the introns and identifying sets of reactions that failed when patient DNA was used as the template. DNA from a normal individual was included in the analysis for comparison. Amplification with primer combinations HIn4 F4-R5 to HIn5 F1-R2 (Table 3) failed to generate a signal from the patient DNA, whereas the normal DNA generated products of the expected size, indicating that the intron 4 breakpoint was between 6.3 to 9.3kb upstream of exon 5, while the intron 5 breakpoint was more than 3.7 kb downstream of that exon Subsequent amplification of patient DNA with HIn4 F4 and the reverse primer targeted to exon 6 (HEx6ROuter) generated an amplicon of about 5.4 kb, while long range PCR of the normal dystrophin gene using these primers generated a product in excess of 16 kb (Fig 2a). Although efficient amplification of the patient-specific amplicon was achieved with both the TAKARA LA and GC buffers, use of the later buffer improved amplification of the larger dystrophin gene product. The 5.4 kb patient-derived amplicon was used as a DNA sequencing template with primers shown in Table 4. Comparison to the reference sequence NG_012232.1 showed that the entire genomic deletion in this patient was 10,864 bp, with two ‘T’s common to introns 4 and 5 at the breakpoint junction (Fig 2b). The position of the 10.8kb genomic deletion is shown, relative to two other exon 5 deletions that were reported to lead to DMD [13] (Fig 2c).

**Fig 3:**
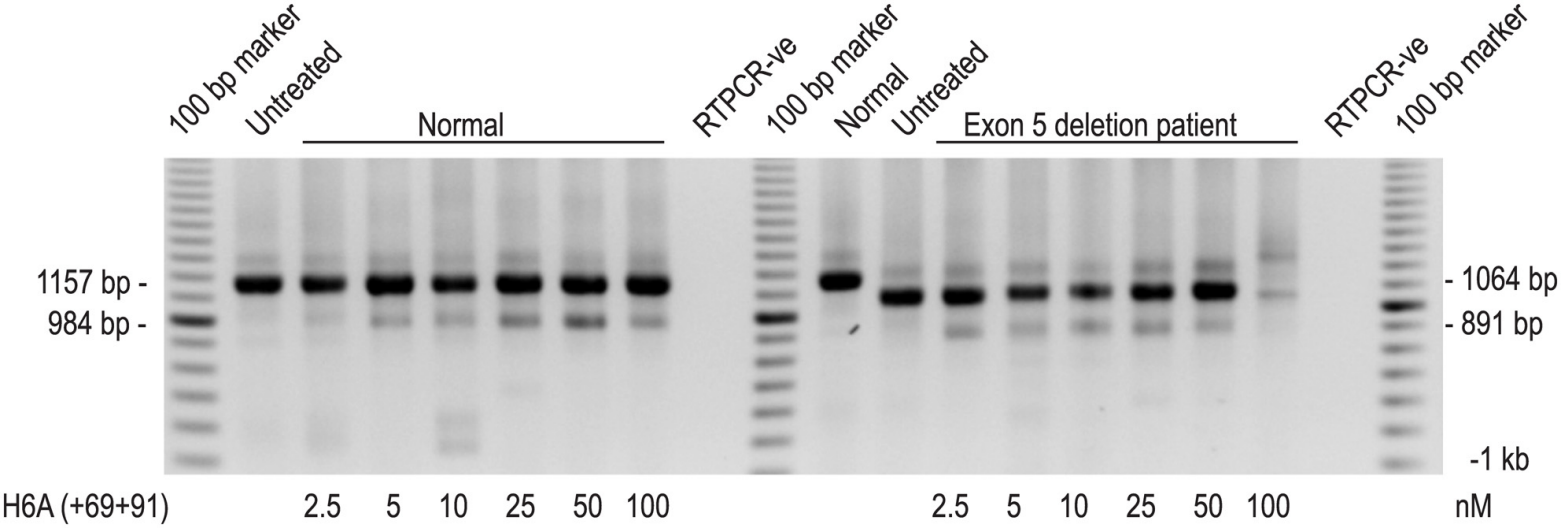
Antisense oligomer induced skipping of *DMD* exon 6 in patient and normal myogenic cells. Normal and *DMD exon 5 deletion* patient fibroblasts, forced into the myogenic lineage by transfection with the MyoD expressing adenovirus, were transfected with an AO designed to excise *DMD* exon 6. Dystrophin exons 1–10 were amplified from RNA prepared from control and oligomer (H6A(+69+91))-treated normal and patient RNA samples by nested RT-PCR. The full-length amplicon is 1157 bp, the amplicon derived by RT-PCR of patient RNA, missing exon 5, is 1064 bp. The induced amplicons missing exon 6 (normal) are 984 bp and 891 (patient). The RT-PCR generating the amplicons from the 100 nM transfection of patient cells was not efficient due to low RNA yields, but both full-length and skipped products are evident.

The predominant amplicon after RT-PCR of patient muscle RNA across *DMD* exons 1–10 was missing only exon 5, and there was no evidence of any *DMD* transcripts missing exon 6 in the patient cells (Fig 3). Normal and patient fibroblasts, forced into the myogenic lineage after transfection with the adenovirus construct expressing MyoD, were transfected with an AO designed to excise *DMD* exon 6 to ascertain if recognition of the exon by the splicing machinery was compromised by the patient deletion. As shown in Fig 3, the efficiency of exon 6 skipping after transfection was similar in both cell strains.

### Induction of an exon 5 deletion dystrophin isoform in wild type mice

**Fig 4:**
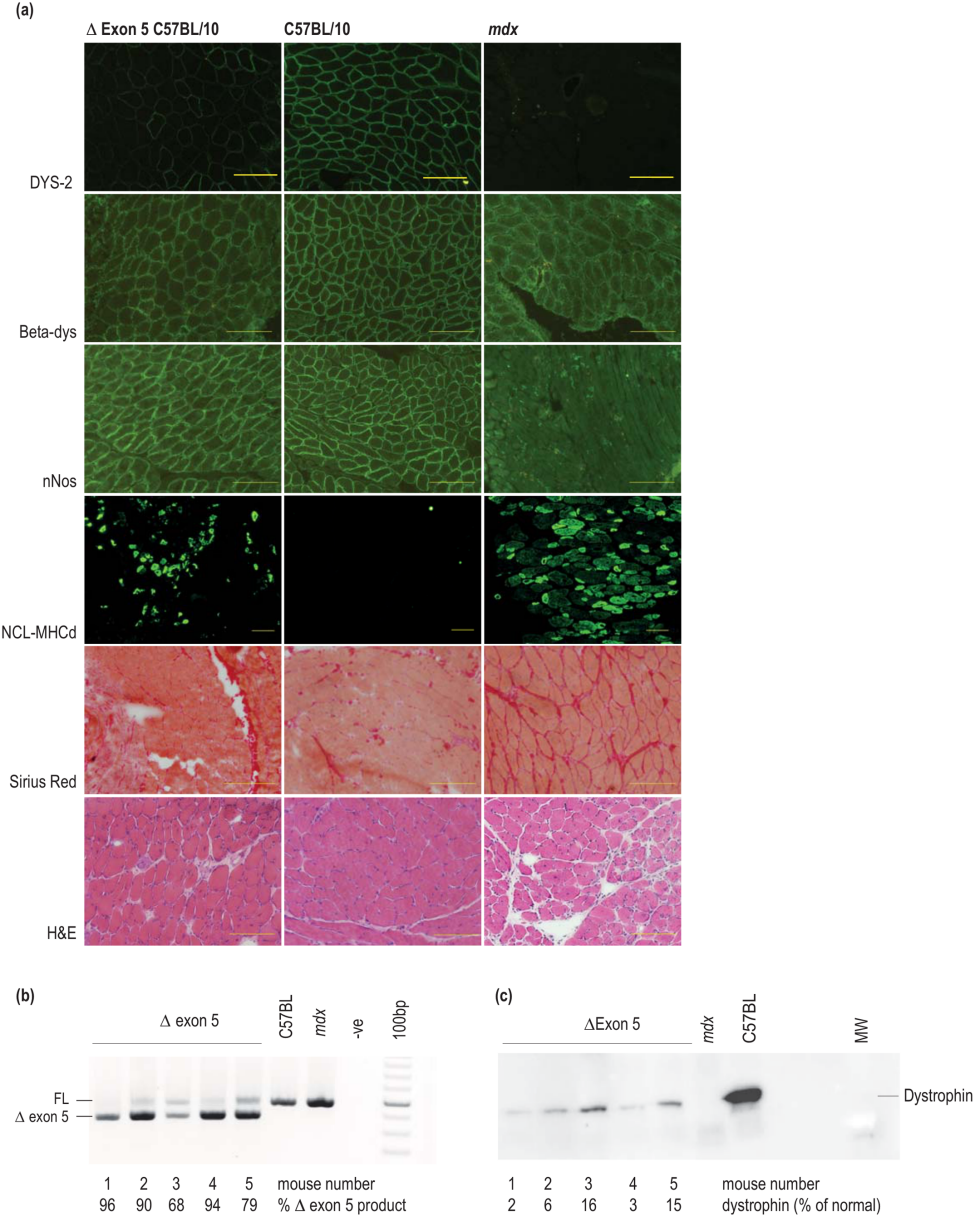
AO-mediated induction of *Dmd* exon 5 deletion dystrophin isoform in wild type mice. **(a)** Cryosections from diaphragm of *5-PPMOk* and sham treated C57BL10 mice, and sham treated *mdx* mice were stained to reveal dystrophin carboxy terminus (NCL DYS-2), β-dystroglycan (β-Dys), neuronal nitric oxide synthase (nNOS) and developmental myosin heavy chain (NCL-MHCd). Picro sirius red was used to stain collagen, as an indicator of fibrosis, and muscle architecture is revealed by hematoxylin and eosin (H&E) staining. (Bar = 200μm) (b) RT-PCR across *Dmd* exons 1–7 on RNA extracted from diaphragm of *5-PPMOk (n = 5)* and sham treated C57BL10 mice, and sham treated *mdx* mice. The percentage of induced amplicon (exon 5 deleted) as a percentage of the total transcript product is indicated. (c) Western blot on protein extracts (7.5μg total protein per lane) from diaphragm of *5-PPMOk (n = 5)* and sham treated C57BL10 mice, and sham treated *mdx* mice. Dystrophin was detected using NCL-Dys2 (Novocastra Laboratories) and Western Breeze (Life Technologies/Thermo Fisher Scientific). The percentage of dystrophin, relative to the wild type control, was assessed by densitometry after normalization to myosin heavy chain expression.

An AO (2’-O-methyl modified bases on a phosphorothioate backbone), designed to anneal to mouse dystrophin exon 5 bases 20 to 49, was identified in a panel of overlapping oligomers as efficiently skipping this exon after *in vitro* transfection in primary mouse myogenic cells (data not shown). This sequence was synthesized as a PMO conjugated to a cell penetrating peptide for *in vivo* studies in wild type mice, with the aim to generate a transient *Dmd* exon 5 deletion *in vivo* model. A dystrophic phenotype, induced by skipping of *Dmd* exon 5 was evident in the diaphragms, although the dystrophic pathology was quite as marked as for the sham treated *mdx* mice. Immunostaining using the antibody, NCL-DYS2 to detect the carboxy terminus of dystrophin reflected patchy and greatly reduced staining in the diaphragm of *5-PPMOk* treated C57BL/10 mice, compared to sham treated control C57BL/10 mice (Fig 4a, top row), and western blotting indicated that dystrophin levels were reduced to less than 10% of wild-type levels (data not shown). The reduced dystrophin staining was associated with reduced β-dystroglycan and nNOS expression in diaphragm of treated mice (Fig 4a, left panel), compared to levels in sham-treated control C57BL/10 mice (Fig 4a, central panel), whereas expression in *mdx* mouse diaphragm is very low. A modest degree of muscle regeneration in the diaphragm from *5-PPMOk-* treated mice is indicated by the presence of developmental myosin heavy chain (dMHC) in muscle fibres (Fig 4a). Neither muscle regeneration nor fibrosis is evident in diaphragm from sham-treated C57BL10 mice, whereas abundant dMHC staining, fibrosis revealed by Picro-sirius Red staining and central nucleation is apparent in the *5-PPMOk* treated C57BL10 and in the sham treated *mdx* mice, and is consistent with a moderate dystrophic muscle phenotype.

RT-PCR across *Dmd* exons 1–7 on RNA extracted from diaphragm of *5-PPMOk (n = 5)* and sham treated C57BL10 mice, and sham treated *mdx* mice showed efficient skipping of exon 5, varying from 68–96% of the total *Dmd* transcript product, as determined by densitometry. (Fig 4b). Western blot analysis on protein extracts from diaphragm of *5-PPMOk* treated C57BL10 mice showed greatly reduced dystrophin expression, relative to the sham-treated wild type control (Fig 4c). The percentage of dystrophin, relative to the wild type control, was assessed by densitometry after normalization to myosin heavy chain expression and correlated with analysis of the *Dmd* transcript shown in Fig 4b.

## Discussion

The dystrophin reading frame rule holds true for over 90% of DMD and BMD deletion cases, [28] [29] determined by DNA molecular diagnoses [30], [31]. Detailed examination of causative mutations in cases that are ‘reading frame exceptions’ have revealed several mechanisms that account for a discrepancy between *DMD* genotype and clinical phenotype. In-frame exonic deletions may result in a severe phenotype if the deletion involves more than 25 exons in [32]) or results in the loss of a crucial functional motif, eg that encoding the beta-dystroglycan binding domain or the first 20 exons [32]. Protein truncating mutations in the 5’ region can lead to re-initiation of translation in exons 5, 6 or 8 and synthesis of a functional dystrophin [11] [33]. Secondary RNA processing events, not apparent by analysis of the DNA, may influence exon selection and processing of the transcript. Nonsense mutations in some of the in-frame exons encoding the dystrophin rod domain manifest with a milder than expected phenotype due to natural skipping of the mutated and splice compromised exon [34] [35].

Single exon deletions in the *DMD N*-terminal region are relatively uncommon, with 8 entries in the Leiden database (http://www.dmd.nl) describing deletion of only exon 5. Although clinical details on these patients are minimal, three were listed as having DMD and the remaining five were recorded as having Intermediate MD. Interestingly, there were seven entries for a duplication of *DMD* exon 5, with five records reporting a BMD phenotype and one entry each listed as having DMD or Intermediate MD.

Detailed *DMD* transcript studies by Gualandi and colleagues reported that exon 6, present in the genomes of their exon 5 deletion patients was absent from the dystrophin mRNA, indicating that these genomic deletions compromised exon 6 recognition by the splicing machinery [13]. The loss of exons 5 and 6 from the *DMD* transcript would disrupt the dystrophin reading frame, consistent with a severe pathology. The patient that we describe had a genomic deletion of only exon 5, and RNA analysis indicated that only that exon was missing from the *DMD* mRNA. We hypothesize that the size and/or location of this particular in frame deletion, 10.8kb compared to the 7.78 and 3.84 kb deletions identified in the other exon 5 deletion patients [13], may have contributed to the processing of dystrophin exons during splicing. Gualandi and colleagues [13] proposed that a 2.1 kb block of sequence, immediately upstream of exon 6 and common to both *DMD* exon 5 deletion patients, influences splice site selection and the novel fusion introns favour exon 6 skipping. This possibility no longer seems likely since the deletion breakpoint in our patient is located between those in the DMD exon 5 deleted patients reported by Gualandi and colleagues [13].

It is possible that novel sequence at the 10.8 kb deletion breakpoint may have strengthened exon 6 recognition compared to other *DMD* exon 5 deletions. Clearly, genomic rearrangements and the subsequent fusion of normally non-contiguous sequences can have unexpected consequences [36]. However, we were able to demonstrate that AO induced skipping of exon 6 in myogenic cells from our exon 5 deletion patient occurred at a similar efficiency to that induced in normal myogenic cells, suggesting that this 10.8 kb deletion does not compromise dystrophin exon 6 recognition and processing. Since the deletion of exon 5 does not disrupt the reading frame, but nevertheless the relative amounts of dystrophin were low as assessed by western blotting and immunofluorescence, it must be assumed that the quality of a dystrophin isoform lacking the amino acids encoded by exon 5 is compromised.

In order to further explore the functionality of the dystrophin isoform, missing the domain encoded by exon 5, we used an AO designed to exclude exon 5 from the mature *Dmd* transcript in wild-type mice, essentially creating a transient dystrophinopathy mouse model. Dystrophin expression and localisation, and muscle architecture in the patient’s muscle is reflected by the analysis of *5-PPMOk* treated mouse diaphragm. Dystrophin expression is greatly reduced, beta-dystroglycan and nNOS are somewhat reduced, developmental myosin expression is readily detectable in numerous muscle fibres and the muscle shows increased collagen staining, fibre size variation and the presence of central nuclei, indicating active muscle regeneration. These features are not quite as marked as those in *mdx* mouse diaphragm, in which dystrophin is undetectable apart from occasional revertant fibres. Although *Dmd* exon 5 is small and in-frame, exclusion of this exon removes the entire second actin binding sequence and appears to severely compromise function of the dystrophin isoform, leading to a more severe muscle phenotype.

The three dystrophin primary actin binding sites (ABS 1-2-3), specifically amino acids 18–27, 88–116 and 131–147, are encoded by *DMD* exons 2, 5 and 6. Since pathogenic missense mutations have been identified in this region: L54R in exon 3, A168D and A171P in exon 6, and Y231N in exon 8, the loss of in-frame dystrophin exon 5 encoding the entire ABS2 could impact on dystrophin binding to F actin [37].

The issue is further complicated when other in frame deletion dystrophin isoforms are considered. The deletion of dystrophin exons 3–9 removes ABS 2 and 3, but caused only mild symptoms in a patient diagnosed with BMD after the age of 60 years [38]. Although this suggests that only ABS1 is needed for functional actin binding, Amann and colleagues [39–41] reported a cluster of basic repeats in the rod domain, amino acids 1416–1880 encoded by exons 31 to 40 also bound F-actin, and this secondary binding site contributed to the milder phenotype. Since the secondary binding domain would also be present in the *DMD* exon 5 deleted individuals, it is conceivable that the spatial disruption of the remaining ABS 1 and 3 compromises actin binding.

Multi-exon frame-shifting deletions in the 5’ end of *DMD* are more common than small in-frame deletions, and this is most likely due to intron length and also the number of SINES, LINES, Alu repeats and microsatellite blocks [42]. However, the few reports of single in-frame exon deletions in the primary actin-binding domain indicate that such changes have variable consequences. Two brothers with a deletion of *DMD* exon 3, showed early onset dystrophy and loss of ambulation, but stabilised in early adulthood and did not have respiratory or cardiac insufficiency [29]. A Japanese male, diagnosed as autistic at age 3 years was subsequently found to have a *DMD* deletion of exon 4. He was reported to have mild pseudo-hypertrophy, modestly elevated CK, but with otherwise normal muscle strength [43, 44]. His three affected brothers were found to have similar severe mental retardation and also did not exhibit any muscle weakness. One can only speculate if the autism was related to the dystrophin isoform in this one family.

The dystrophin isoform encoded by the transcript missing exon 5 appears to have reduced function, and therefore clinical application of exon 5 skipping to overcome intra-exonic mutations may be of limited benefit. Consequently, the simplest exon skipping strategy, that is, skipping exon 5 may not offer the best outcome for patients with intra-exonic mutations in this region of the gene. A deletion of *DMD* exons 3–9 is reported to be associated with a mild BMD phenotype [38], and further investigations on patients with other in-frame deletions in the *DMD* amino terminal region may help to define better functional dystrophin isoforms that could be induced by AO mediated exon selection.
